# The Impact of ^131^I-Metaiodobenzylguanidine as a Conditioning Regimen of Tandem High-Dose Chemotherapy and Autologous Stem Cell Transplantation for High-Risk Neuroblastoma †

**DOI:** 10.3390/children10121936

**Published:** 2023-12-18

**Authors:** Hyun Jin Park, Jung Yoon Choi, Bo Kyung Kim, Kyung Taek Hong, Hyun-Young Kim, Il Han Kim, Gi Jeong Cheon, Jung-Eun Cheon, Sung-Hye Park, Hyoung Jin Kang

**Affiliations:** 1 Department of Pediatrics, Seoul National University College of Medicine, Seoul 03080, Republic of Korea; fionajin05@snu.ac.kr (H.J.P.); hongkt@snu.ac.kr (K.T.H.); 2 Seoul National University Cancer Research Institute, Seoul 03080, Republic of Korealarrycheon@snu.ac.kr (G.J.C.); 3 Department of Pediatric Surgery, Seoul National University College of Medicine, Seoul 03080, Republic of Korea; 4 Department of Radiation Oncology, Seoul National University College of Medicine, Seoul 03080, Republic of Korea; 5 Department of Nuclear Medicine, Seoul National University College of Medicine, Seoul 03080, Republic of Korea; 6 Department of Radiology, Seoul National University College of Medicine, Seoul 03080, Republic of Korea; cheonje@snu.ac.kr; 7 Department of Pathology, Seoul National University College of Medicine, Seoul 03080, Republic of Korea; shparknp@snu.ac.kr; 8 Wide River Institute of Immunology, Hongcheon 25159, Republic of Korea

**Keywords:** neuroblastoma, autologous stem cell transplantation, chemotherapy, pediatrics

## Abstract

Background: The optimal conditioning regimen of tandem high-dose chemotherapy (HDC) and autologous stem cell transplantation (ASCT) for high-risk neuroblastoma (HR-NBL) has not been established. The efficacy of ^131^I-MIBG therapy is under exploration in newly diagnosed HR-NBL patients. Here, we compared the outcomes of tandem HDC/ASCT between the ^131^I-MIBG combination and non-MIBG groups. Methods: We retrospectively analyzed the clinical data of 33 HR-NBL patients who underwent tandem HDC/ASCT between 2007 and 2021 at the Seoul National University Children’s Hospital. Results: The median age at diagnosis was 3.6 years. ^131^I-MIBG was administered to 13 (39.4%) of the patients. Thirty patients (90.9%) received maintenance therapy after tandem HDC/ASCT, twenty-two were treated with isotretinoin ± interleukin-2, and eight received salvage chemotherapy. The five-year overall survival (OS) and event-free survival (EFS) rates of all patients were 80.4% and 69.4%, respectively. Comparing the ^131^I-MIBG combined group and other groups, the five-year OS rates were 82.1% and 79.7% (*p* = 0.655), and the five-year EFS rates were 69.2% and 69.6% (*p* = 0.922), respectively. Among the adverse effects of grade 3 or 4, the incidence of liver enzyme elevation was significantly higher in the non-^131^I-MIBG group. Conclusions: Although tandem HDC/ASCT showed promising outcomes, the ^131^I-MIBG combination did not improve survival rates.

## 1. Introduction

Neuroblastoma (NBL) is the most common extracranial solid tumor in childhood. It has been classified into low-, intermediate-, and high-risk groups based on diagnostic age, histology, stage, and molecular markers. Pediatric oncologist groups, including the International Neuroblastoma Risk Group, regularly update the classification. Because the prognosis and treatment response are markedly different between risk groups, risk-adjusted therapy has been applied [1,2]. Although the treatment outcome of NBL has improved due to multimodal and intensive treatment, high-risk NBL (HR-NBL) still has a poor prognosis. The five-year overall survival (OS) in HR-NBL is approximately 50% in the latest reports [3,4,5].

High-dose chemotherapy (HDC) and autologous stem cell transplantation (ASCT) are standard treatments in HR-NBL, and to enhance the effect, diverse studies on HDC/ASCT in HR-NBL are ongoing [3,6,7]. Regarding intensification, Park et al. reported that tandem HDC/ASCT was superior to single HDC/ASCT in terms of three-year event-free survival (EFS), especially when combined with immunotherapy [8]. A phase III clinical trial was conducted to optimize the regimen for HDC/ASCT by comparing busulfan and melphalan (BuMel) with melphalan, etoposide, and carboplatin (MEC). The results showed that BuMel prolonged three-year EFS compared with MEC and caused fewer grade 3–4 adverse events but more frequent veno-occlusive disease (VOD) [9]. However, there is no consensus on a tandem HDC regimen. Park et al. used thiotepa/cyclophosphamide (ThioCy) and MEC for the first and second HDCs [8]. Pasqualini et al. used high-dose thiotepa and BuMel as a tandem HDC regimen in HR-NBL [10].

Based on the concept that most NBL accumulates meta-iodobenzylguanidine (MIBG), MIBG radiolabeled with ^131^iodine (^131^I-MIBG) has been used for targeted radiotherapy [11]. ^131^I-MIBG was initially used in relapsed/refractory NBL, and due to its antitumor effects, attempts have been made to apply ^131^I-MIBG to newly diagnosed HR-NBL and combine ^131^I-MIBG with other conventional chemotherapy [11,12]. Hamidieh et al. added ^131^I-MIBG to HDC/ASCT, but the three-year EFS did not differ significantly between the groups [13]. The ^131^I-MIBG combination was successfully performed without a marked increase in toxicity, but the efficacy and optimal indication have not been determined [11,12]. Furthermore, as the production of ^131^I-MIBG has been reduced in Korea, the use of ^131^I-MIBG is becoming increasingly difficult.

Against this background, we retrospectively analyzed the outcome of the ^131^I-MIBG combination in tandem with HDC/ASCT in HR-NBL patients. We conducted tandem HDC/ASCT using a consistent conditioning regimen, topotecan-thiotepa-carboplatin (TTC) for the first HDC/ASCT and MEC for the second HDC/ASCT. Additionally, we incorporated ^131^I-MIBG therapy one month before the second HDC/ASCT, starting in 2013 when ^131^I-MIBG therapy was introduced at our center. After tandem HDC/ASCT, we implemented radiation therapy and selectively administered maintenance therapy according to the treatment response. Through this study, we aim to evaluate the efficacy and feasibility of ^131^I-MIBG therapy as a conditioning regimen for tandem HDC/ASCT for HR-NB, focusing specifically on its preemptive use rather than as a salvage treatment.

## 2. Methods

### 2.1. Patients

We reviewed clinical data of 33 patients diagnosed with HR-NBL who underwent tandem HDC/ASCT from 2007 to 2022. We included patients who had completed both the first and second HDC/ASCT with TTC/MEC regimens and excluded patients who did not receive the second planned HDC/ASCT due to disease progression or complications. The indication for tandem HDC/ASCT comprised patients aged ≥ 1 year at diagnosis with International Neuroblastoma Staging System (INSS) stage 4, patients aged < 1 year at diagnosis with INSS stage 4 and with amplified *MYCN* proto-oncogene, bHLH transcription factor (*MYCN*), and patients with INSS stage 3 at any age and with *MYCN* amplification [14].

### 2.2. Assessment of Disease Extent and Response Criteria

The tumor extent was evaluated using computed tomography (CT) or magnetic resonance imaging, technetium-99m bone scintigraphy, ^18^F-fluoro-deoxy-D-glucose positron emission tomography (FDG-PET)/CT, and bilateral bone marrow examination. ^123^I-MIBG scans were performed to evaluate MIBG uptake in the tumor. *MYCN* amplification was identified by fluorescence in situ hybridization of tumor tissues.

Responses were evaluated every three cycles of induction chemotherapy, after surgical resection, before the first and second HDC/ASCT, every three months for the first year after the second HDC/ASCT, every four months for the second year, every six months for the third year, and every year thereafter. Treatment response was assessed according to the International Neuroblastoma Response Criteria [15].

### 2.3. Pretransplant Treatment

Induction chemotherapy consisted of cisplatin (60 mg/m^2^), etoposide (200 mg/m^2^), adriamycin (30 mg/m^2^), and cyclophosphamide (CPM, 60 mg/kg), based on CCG 321-P2 [16]. When the response to CCG 321-P2 was poorer than the partial response (PR), we changed the regimens to modified CCG-ICE (ifosfamide, 6000 mg/m^2^; carboplatin, 700 mg/m^2^; and etoposide, 400 mg/m^2^) or TCE (CPM, 1250 mg/m^2^; topotecan, 5 mg/m^2^; and etoposide, 300 mg/m^2^) [17,18]. At least six cycles of pretransplant chemotherapy were performed before the first HDC/ASCT, and if possible, surgical resection was performed. After confirming the absence of bone marrow involvement, CPM (3000 mg/m^2^) and etoposide (450 mg/m^2^) were administered for peripheral stem cell mobilization (Table 1). Granulocyte colony-stimulating factor (G-CSF) was administered on day 7 of CPM/etoposide chemotherapy.

### 2.4. Tandem HDC/ASCT

The regimen for the first HDC consisted of topotecan (10 mg/m^2^), thiotepa (900 mg/m^2^), and carboplatin (1500 mg/m^2^) (TTC). The regimen for the second HDC consisted of melphalan (210 mg/m^2^), etoposide (800 mg/m^2^), and carboplatin (1400 mg/m^2^) (MEC). The carboplatin dose in the ^131^I-MIBG combined group was reduced to 1200 mg/m^2^ (mMEC + ^131^I-MIBG) (Table 1). We added ^131^I-MIBG to the second HDC in 2013. In the mMEC + ^131^I-MIBG protocol, ^131^I-MIBG was administered on day 21 of the second ASCT and the dose of MIBG treatment was 12 mCi/kg, except in one patient with 17 mCi/kg. The minimal interval between each ASCT was 12 weeks, and if complications occurred following the first HDC/ASCT, we performed a second HDC/ASCT after the complication had resolved completely. All patients received G-CSF from day 1 of autologous peripheral stem cell infusion to recovery of neutrophils >3000/μL or 1000/μL for 3 consecutive days.

### 2.5. Post-HDC/ASCT

One month after the second HDC/ASCT, the patient received radiation therapy in the primary tumor bed. If a residual tumor was suspected in the first image evaluation, three months after the second HDC/ASCT, a second-look surgery was performed to determine whether tumor cells remained. In case of complete response (CR), we administered immunotherapy with interleukin-2 (IL-2) and isotretinoin (ITT) for two years as maintenance therapy. In other cases, we administered salvage intensification chemotherapy, cyclophosphamide, and topotecan.

### 2.6. Evaluation of Adverse Effects

Acute toxicities during HDC/ASCT were monitored according to the Common Terminology Criteria for Adverse Events (version 4.0) of the US National Cancer Institute. VOD was evaluated using the Modified Seattle Criteria, and thrombotic microangiopathy (TMA) was evaluated using criteria from the International Working Group [19].

### 2.7. Survival Analysis and Statistics

Differences in continuous variables were measured using the Student’s *t*-test or Mann-Whitney test. Differences in categorical variables were measured using the chi-square test or Fisher’s exact test. The EFS was calculated from the date of the second ASCT to the date of relapse, progression, secondary malignancy, or death, whichever occurred first. The OS was calculated from the date of the second ASCT to death from any cause. We analyzed patients for whom no clear events or deaths, including cases of follow-up loss, were reported, considering them censored at the last follow-up visit. Survival rates and standard errors were estimated using the Kaplan-Meier method. Differences in survival rates between the two groups were compared using the log-rank test. *p*-values < 0.05 were considered significant.

## 3. Results

### 3.1. Patients’ Characteristics

A total of 33 HR-NBL patients were retrospectively analyzed, and the patients’ characteristics are summarized in Table 2. The median age at diagnosis was 3.6 years (range, 4 months to 13.6 years), and the median follow-up duration from diagnosis was 9.3 years (range, 1.1–16.1). The youngest patient was four months old at diagnosis and was classified as having INSS stage 4S, but had an *MYCN*-amplified tumor. The most frequent primary site was the retroperitoneum, including the adrenal gland (29/33, 87.9%), and other primary lesions were in the pelvic cavity, paraspinal area, posterior mediastinum, and left upper abdominal cavity. All patients had metastatic tumors, and the most common metastatic sites were the lymph nodes (27/33, 81.8%), bone marrow (21/33, 63.6%), and bone (21/33, 63.6%). In terms of histologic classification, if the primary tumor biopsy was limited or performed after chemotherapy, these cases were categorized as “unknown” subtype of neuroblastoma.

### 3.2. Pretransplant Chemotherapy

The median number of pretransplant chemotherapy cycles was 7 (range, 5–15), and the median duration of pretransplant chemotherapy was six months (range, 3–14). Seven patients (21.2%) changed regimens during induction; six of these changes were due to residual or progressive disease, and one was due to drug-related toxicity and cardiomyopathy. The patient with cardiomyopathy completed the treatment without aggravation after excluding the cardiotoxic drug adriamycin. All patients had neutropenic fever, and other toxicities during pretransplant chemotherapy were manageable. Patients achieved CR (4/33, 12.1%) or PR (29/33, 87.9%) before the first HDC/ASCT.

### 3.3. Tandem HDC/ASCT

For the first and second HDC/ASCT, the median infused CD34+ dose was 5.28 (range, 0.99–12.92) × 10^6^ cells/kg and 4.96 (range, 1.175–12.92) × 10^6^ cells/kg, respectively. The mean infused CD34+ doses in the second HDC/ASCT of the MEC and mMEC + ^131^I-MIBG groups were 6.54 ± 3.20 × 10^6^ cells/kg and 4.25 ± 2.34 × 10^6^ cells/kg (*p* = 0.034), respectively. Except for one patient with therapy-related mortality during the second ASCT, all patients had engraftment of neutrophils and platelets. For the first ASCT, all patients had neutrophil and platelet engraftment on the median of day 10 (range, 9–13) and day 13 (range, 10–18), respectively. After the second ASCT, the median times for neutrophil and platelet engraftment were 10 days (range, 8–13) and 14 days (range, 9–27), respectively. Comparing the engraftment time between the MEC and mMEC + ^131^I-MIBG groups, the mean neutrophil engraftment duration for each group was 10.42 ± 1.17 and 9.69 ± 0.48 (*p* = 0.022), respectively. The median interval between the first and second ASCT was 98 days (range, 82–275). One patient delayed the second HDC/ASCT for 275 days due to cytomegalovirus retinitis. In the mMEC + ^131^I-MIBG group, the median dose of ^131^I-MIBG was 12.2 mCi/kg (range, 10.9–17.6). One patient received 17.6 mCi/kg of ^131^I-MIBG as a myeloablative dose targeting residual tumor, and the dose for the other patients was set at 12 mCi/kg.

### 3.4. Post-Consolidation Therapy

After the second HDC/ASCT, 26 patients (78.8%) received radiation therapy to the primary tumor bed, which was incompletely resected by surgery. The median interval between the second ASCT and radiation therapy was 46.5 days (range, 31–73 days). The median dose of radiotherapy to the tumor bed was 16.5 Gy (range, 12–27). Second-look surgery was performed on eight patients and a CT-guided biopsy was performed on one patient. Seven of them were pathologically confirmed to have a residual tumor.

Among the 30 patients, excluding three patients with therapy-related mortality (TRM) or who were lost to follow-up immediately after the second ASCT, eight patients (8/30, 26.7%) with pathologically confirmed residual tumor or radiologically progressive disease received salvage chemotherapy as maintenance therapy, and the median number of chemotherapy cycles was 22 (range, 3–50). Of the remaining 22 patients (22/30, 73.3%), 3 (10%) received ITT, and 19 (63.3%) received IL-2 and ITT. Between the MEC and mMEC + ^131^I-MIBG groups, the ratio of maintenance therapy types was not significantly different (Table 2).

### 3.5. Toxicity and Complications

Table 3 lists acute and chronic complications during the first and second HDC/ASCT procedures. The most common adverse effect was febrile neutropenia in the first and second HDC/ASCT; however, except for one patient with respiratory syncytial virus (RSV)-associated pneumonia, there were no documented bacterial, fungal, or viral infections. Between the two groups, grade 3 or 4 liver enzyme elevations were significantly more frequent in the MEC group (*p* < 0.001).

There were two TRMs during the second HDC/ASCT. One case in the MEC group was caused by acute renal failure and sudden cardiac arrest; no evidence of VOD was found. The other case in the mMEC + ^131^I-MIBG group was caused by RSV-associated pneumonia, identified in the bronchoalveolar lavage specimen. Although ribavirin was administered, progressive pneumonitis with fever was not controlled. One patient was treated for VOD during the second HDC procedure, which was manageable. Two patients were diagnosed with TMA after the second HDC procedure and underwent plasmapheresis.

Among the 26 patients monitored for over one year after completing treatment, hypothyroidism and growth failure occurred in four and eight patients, respectively. There were no significant differences in hypothyroidism or growth failure incidence between the MEC and mMEC + ^131^I-MIBG groups (*p* = 0.591, 0.667, respectively).

### 3.6. Relapse/Progression and Secondary Malignancy

Relapse or progression occurred in nine patients (27.3%) at a median of 11 months (range, 2–74) after the second ASCT. Three patients were lost to follow-up, five died, and one survived without disease after salvage chemotherapy. Six patients were in the MEC group (6/20, 30.0%) and three were in the mMEC + ^131^I-MIBG group (3/13, 23.1%) (*p* = 1.00).

One patient was diagnosed with therapy-related myelodysplastic syndrome 6.8 years after the second HDC/ASCT. Renal cell carcinoma, squamous cell carcinoma, and rhabdomyosarcoma occurred in three patients after 7.1, 3.8, and 12.0 years, respectively. All four patients belonged to the MEC group.

### 3.7. Tumor Response and Survival

**Figure 1:**
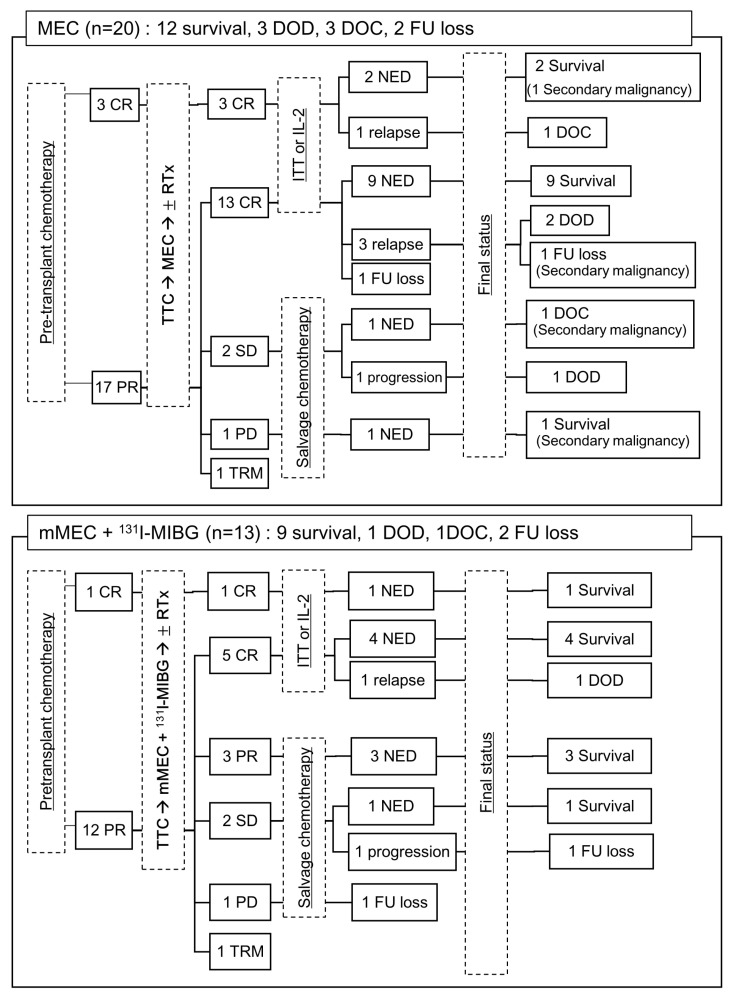
Treatment responses of all involved patients. CR, complete response; PR, partial response; SD, stable disease; PD, progressive disease; FU, follow-up; TRM, therapy-related mortality; NED, no evidence of disease; DOC, died of complication; DOD, died of disease; TTC, Topotecan/Thiotepa/Carboplatin; MEC, Melphalan/Etoposide/Carboplatin; ^131^I-MIBG, ^131^Iodine-metaiodobenzylguanidin; RTx, radiation therapy; ITT, isotretinoin; IL-2, Interleukin-2.

The overall treatment course and responses to each treatment are shown in Figure 1. Four patients with CR after induction chemotherapy continued CR after tandem HDC/ASCT. One patient experienced disease recurrence and died from complications after chemotherapy. Of the twenty-nine patients with PR from induction chemotherapy, there were eighteen with CR, three with PR, four with stable disease, two with progressive disease, and two with TRM after tandem HDC/ASCT. Among the patients who received salvage chemotherapy, five were alive without disease.

**Figure 2:**
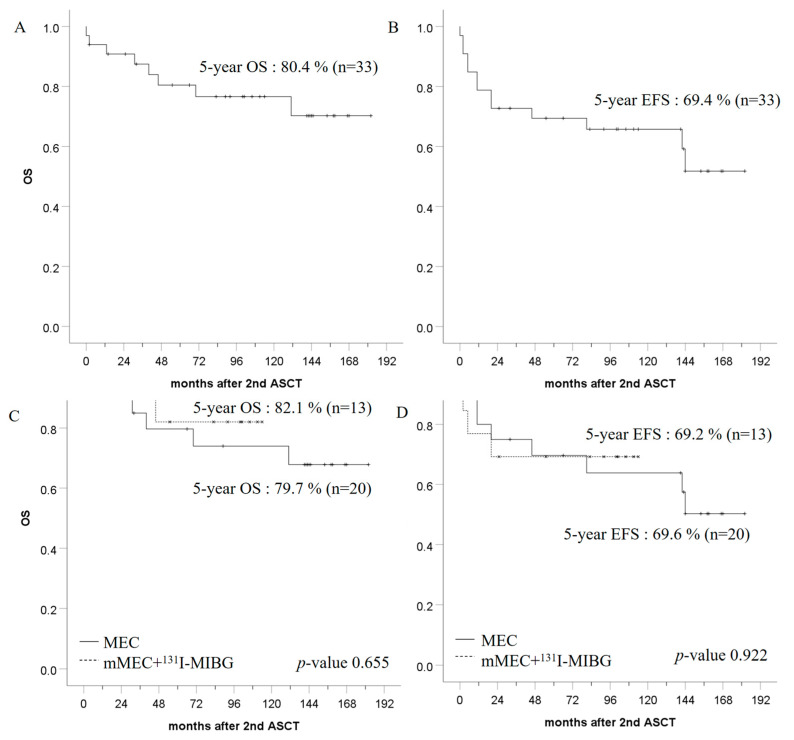
Patients’ survival. (**A**) Overall survival (OS) of all the patients. (**B**) Event-free survival (EFS) of all the patients. (**C**) OS in MEC and mMEC + ^131^I-MIBG groups. (**D**) EFS in MEC and mMEC + ^131^I-MIBG groups.

Figure 2 shows the OS and EFS rates for each group. The five-year OS and EFS rates in all patients were 80.4% and 69.4%, respectively. Comparing the ^131^I-MIBG combined group and the other groups, the five-year OS rates were 82.1% and 79.7% (*p* = 0.655), and the five-year EFS rates were 69.2% and 69.6% (*p* = 0.922), respectively (Figure 2C,D). Reanalyzing survival rates by risk factors, the five-year OS rates of *MYCN*-positive and *MYCN*-negative patients were 55.6% and 90.2% (*p* = 0.019), and the five-year EFS rates of *MYCN*-positive and *MYCN*-negative patients were 44.4% and 78.3% (*p* = 0.064), respectively. The five-year OS and five-year EFS rates for stages 3 and 4 were 71.4% and 82.9% (*p* = 0.531) and 71.4% and 69.0% (*p* = 0.772), respectively.

## 4. Discussion

In this study, we evaluated the treatment outcome of tandem HDC/ASCT with a uniform conditioning regimen, TTC/MEC, and the efficacy of the ^131^I-MIBG combination as a conditioning regimen. After completion of consolidation therapy, we administered maintenance therapy and stratified the intensity based on treatment response. The results were favorable, with five-year OS and EFS rates of 80.4% and 69.4%, respectively. There were two TRMs during the second HDC/ASCT, and other acute toxicities were manageable. The combination of ^131^I-MIBG did not show significant differences in survival rates or major toxicity.

Despite intensive multimodal therapy, HR-NBL is known to have a poor prognosis. Approximately half of NBL cases are refractory or relapse after first-line therapy [3,4]. HDC/ASCT has improved the survival rates of HR-NBL [6,7,20,21]. A randomized trial showed that tandem HDC/ASCT combined with post-consolidative immunotherapy improved the three-year EFS, while cumulative toxicities appeared similar [8]. Still, an optimal regimen for HDC/ASCT has not been established. 

For a single HDC/ASCT, institutions in the USA use MEC widely, while BuMel is used in Europe and the Middle East [9]. Several reports have compared MEC and BuMel, and in a recent randomized phase III trial, BuMel showed better three-year EFS with fewer complications [9,22,23]. For tandem HDC/ASCT, conditioning regimens vary significantly. Park et al. reported that three-year EFS after tandem HDC/ASCT with ThioCy and MEC was 61.6% [8]. Suh et al. used BuMel or MEC for the first HDC and ThioCy + ^131^I-MIBG for the second HDC, and the five-year OS and EFS rates were 79% and 61%, respectively [24]. Lee et al. used MEC and thiotepa-melphalan + ^131^I-MIBG for the first and second HDC, and the five-year OS and EFS were 72.4 and 58.3%, respectively [25]. While a direct comparison is challenging, we obtained comparable outcomes using TTC/MEC when considering the five-year survival rates.

Meanwhile, most NBL cases feature MIBG uptake, and radiolabeled MIBG has long been used as a diagnostic and therapeutic tool [11]. At first, the radioactive target therapeutic, ^131^I-MIBG, was evaluated as monotherapy in relapsed/refractory NBL. The objective response rate ranged from 0% to 66% and definite pain relief was observed, although without objective responses [12]. The efficacy of ^131^I-MIBG therapy in newly diagnosed HR NBL patients is also under evaluation. Hamidieh et al. reported the results of a prospective pilot study comparing ^131^I-MIBG-combined and chemotherapy-only groups. Although ^131^I-MIBG was administered to patients with positive MIBG avidity, the three-year EFS and OS were not statistically different [13]. Reports from Lee et al. and Suh et al. showed that tandem HDC/ASCT concomitant with ^131^I-MIBG did not improve survival rates but only reduced several toxicities [24,25]. The Children’s Oncology Group showed feasibility of ^131^I-MIBG combination prior to myeloablative therapy (BuMel) and a phase III randomized trial (ANBL1531) for ^131^I-MIBG versus no ^131^I-MIBG prior to tandem ASCT is ongoing [26]. In this context, our study aimed to determine whether ^131^I-MIBG combination contributes to HDC/ASCT outcomes, but we did not observe a significant improvement.

Upon considering the reasons why the ^131^I-MIBG combination was not effective in our study, we did not apply the ^131^I-MIBG combination based on initial MIBG avidity. Some patients were not initially evaluated for MIBG avidity because ^123^I-MIBG scans were not possible at the time of diagnosis. Johnson et al. reported that some patients with CR on ^123^I-MIBG scans had residual MIBG-avid lesions on ^131^I-MIBG scans [27]. Based on this, after 2013 when ^131^I-MIBG therapy became available at our institution, we applied a ^131^I-MIBG combined conditioning regimen to all HR-NBL patients subjected to tandem HDC/ASCT, including patients negative for a ^123^I-MIBG scan before the second HDC/ASCT. As a result, two patients were included in the ^131^I-MIBG combination group, even though MIBG-avid lesions were not found on the post-therapy ^131^I-MIBG scan.

Additionally, there has been controversy over the dose of ^131^I-MIBG. Dose escalation appears to improve the response rate in phase I/II studies [28]. Most studies have used 12 mCi/kg ^131^I-MIBG based on a phase I study [29], but dose escalation to 18 mCi/kg combined with myeloablative therapy (MEC) was tolerable in refractory NBL patients [30]. In this study, only one patient who received 17.6 mCi/kg survived without disease. However, we could not evaluate the effectiveness of dose escalation due to the small number of cases. Further studies are needed on the dose escalation of ^131^I-MIBG.

As regulations were tightened to reduce the risk of radiation exposure, the production of radioactive ^131^I-MIBG has declined in Korea, and obtaining ^131^I-MIBG has become difficult in the past five years. Given the current situation, it is important to assess the efficacy of the ^131^I-MIBG combination for HDC/ASCT and determine the optimal indications and dosage for ^131^I-MIBG administration. 

Considering toxicity and complications, the TRM rate (2/33, 5.8%) of the study was similar to that of previous reports: 0–9.3% [8,22,23,31]. In one of the patients with TRM who died of renal failure, the second HDC/ASCT was delayed due to cytomegalovirus retinitis. Despite concerns about complications due to prolonged antiviral therapy, we proceeded with the second HDC/ASCT, due to the presence of definite residual tumor. 

Regarding acute toxicity, we used MEC for the second HDC/ASCT, which was identified as a risk factor for TMA [32,33]. Two cases of TMA occurred after the second HDC/ASCT. In terms of engraftment, despite a higher infused cell dose in the MEC group, the duration of neutrophil engraftment was significantly prolonged. Although liver enzyme elevation was significantly more common in the MEC group, all the cases were manageable and there was no irreversible hepatic failure or significant difference in VOD incidence. 

Among long-term toxicity cases, all four cases of secondary malignancy occurred in the MEC group. The reduction of carboplatin dose can be attributed to a lower incidence of secondary malignancies. However, due to the small sample size, the difference was not statistically significant, and given the significantly shorter follow-up duration in the ^131^I-MIBG group, long-term monitoring is necessary for accurate assessment. 

This study has several differences compared with previous reports. First, we used a uniform conditioning chemotherapy regimen and TTC and MEC for the first and second HDC/ASCT. This can reduce the effects caused by differences in chemotherapy regimens. Second, with the exception of one case, we administered the same dose of MIBG at the same time point. Third, we did not divide the two groups according to the MIBG avidity. Therefore, the bias arising from the difference in MIBG avidity can be reduced. On the other hand, this study also has some limitations. Firstly, the overall number of patients is small, and it is a retrospective analysis. Furthermore, since the two groups were divided based on the treatment timing, it is challenging to overlook the impact of the developments in other adjuvant therapies.

In conclusion, the ^131^I-MIBG combination did not improve the outcome of tandem HDC/ASCT for HR-NBL. However, tandem HDC/ASCT with TTC/MEC regimens showed promising results. A prospective randomized study of ^131^I-MIBG combination in tandem HDC/ASCT is necessary, and efforts to optimize the regimen for tandem HDC/ASCT should continue. As tandem HDC/ASCT showed stronger effects, especially with anti-GD2 antibodies, the direction of further study would be to focus on the feasibility of combinations with newly developed targeted agents.

## Figures and Tables

**Table 1:** Regimen and dosage of chemotherapy.

Regimen	Drug	Dose	Schedule	Total Dose
Pretransplant chemotherapy				
CCG 321P2	Cisplatin	60 mg/m^2^/day	Day 0	60 mg/m^2^
	Etoposide	100 mg/m^2^/day	Day 2, 5	200 mg/m^2^
	Adriamycin	30 mg/m^2^/day	Day 2	30 mg/m^2^
	Cyclophosphamide	30 mg/kg/day	Day 3, 4	60 mg/kg
Modified CCG-ICE	Ifosfamide	1200 mg/m^2^/day	Day 0, 1, 2, 3, 4	6000 mg/m^2^
	Carboplatin	350 mg/m^2^/day	Day 0, 1	700 mg/m^2^
	Etoposide	100 mg/m^2^/day	Day 0, 1, 2, 3	400 mg/m^2^
TCE	Topotecan	1 mg/m^2^/day	Day 0, 1, 2, 3, 4	5 mg/m^2^
	Cyclophosphamide	250 mg/m^2^/day	Day 0, 1, 2, 3, 4	1250 mg/m^2^
	Etoposide	100 mg/m^2^/day	Day 0, 1, 2	300 mg/m^2^
PBSCM				
CPM + VP	Cyclophosphamide	1000 mg/m^2^/day	Day 0, 1, 2	3000 mg/m^2^
	Etoposide	150 mg/m^2^/day	Day 0, 1, 2	450 mg/m^2^
	G-CSF	10 μg/kg	Day 7 to end of PBSCM	
1st HDC				
TTC	Topotecan	2 mg/m^2^/day	Day -8, -7, -6, -5, -4	10 mg/m^2^
	Thiotepa	300 mg/m^2^/day	Day -8, -7, -6	900 mg/m^2^
	Carboplatin	500 mg/m^2^/day	Day -5, -4, -3	1500 mg/m^2^
2nd HDC				
MEC	Melphalan	140 mg/m^2^/day (d-7) 70 mg/m^2^/day (d-6)	Day -7, -6	210 mg/m^2^
	Etoposide	200 mg/m^2^/day	Day -8, -7, -6, -5	800 mg/m^2^
	Carboplatin	350 mg/m^2^/day	Day -8, -7, -6, -5	1400 mg/m^2^
mMEC + ^131^I-MIBG	^131^I-MIBG	12 mCi/kg (11–16.5 mCi/kg)	Day -21	
	Melphalan	140 mg/m^2^/day (d-7) 70 mg/m^2^/day (d-6)	Day -7, -6	210 mg/m^2^
	Etoposide	200 mg/m^2^/day	Day -8, -7, -6, -5	800 mg/m^2^
	Carboplatin	300 mg/m^2^/day	Day -8, -7, -6, -5	1200 mg/m^2^

PBSCM: peripheral stem cell transplantation, HDC: high dose chemotherapy. The chemotherapy dose was reduced for patients younger than one-year-old or less than 10 kg of body weight. Weight-based dose for <one-year-old and <10 kg of body weight, median value of weight-based dose and body surface area-based dose for >one-year-old and <10 kg of body weight.

**Table 2:** Patient characteristics.

Characteristics	MEC (n = 20)	mMEC + MIBG (n = 13)	*p*-Value	Total (n = 33)
Sex, n (%)				
Male	11 (55.0)	9 (69.2)	0.485	20 (60.6)
Female	9 (45.0)	4 (30.8)		13 (39.4)
Age, m, median (range) at diagnosis	38 (4–129)	43 (16–163)	0.984	43 (4–163)
INSS stage, n (%)				
Stage 3	5 (25.0)	2 (15.4)	0.676	7 (21.2)
Stage 4	14 (70.0)	11 (84.6)		25 (75.8)
Stage 4S (+*MYCN* amplification)	1 (5.0)	0 (0.0)		1 (3.0)
*MYCN* amplification, n (%)	6 (30.0)	3 (23.1)	1.000	9 (29.0)
INPC, n (%)				
Unfavorable	7 (35.0)	9 (69.2)	0.156	16 (48.5)
favorable	7 (35.0)	2 (15.4)		9 (27.3)
Unknown	6 (30.0)	2 (15.4)		8 (24.2)
^123^I-MIBG avidity, n (%)				
Yes	5 (25.0)	11 (84.6)	0.004	16 (48.5)
No	1 (5.0)	0 (0.0)		1 (3.0)
Unknown	14 (70.0)	2 (15.4)		16 (48.5)
Primary site, n (%)				
Retroperitoneum	16 (80.0)	13 (100.0)	0.136	29 (87.9)
Others	4 (20.0)	0 (0.0)		4 (12.1)
Disease status before ASCT, n (%)				
CR	3 (15.0)	1 (7.7)	1.000	4 (12.1)
PR	17 (85.0)	12 (92.3)		29 (87.9)
Local RTx, n (%)	17 (85.0)	9 (69.2)	0.393	26 (78.8)
Second-look surgery, n (%)	0 (0.0)	8 (57.1)	<0.001	8 (23.5)
Maintenance therapy total, n (%)	19 (95.0)	11 (78.6)	0.283	30 (90.9)
Salvage chemotherapy, n (%)	3 (15.0)	5 (38.5)	0.522	8 (24.2)
ITT, n (%)	2 (10)	1 (7.7)	1.000	3 (9.1)
IL-2 + ITT, n (%)	14 (70.0)	5 (38.5)	0.073	19 (57.6)
Follow-up duration, m, median (range)	114.0 (18–150)	61.0 (13–83)	0.001	76 (13–150)

CR: complete remission, PR: partial remission, RTx: radiation therapy, INPC: International neuroblastoma pathology classification. One patient diagnosed with Stage 4S was reported *MYCN* amplication-positive. Engraftment duration: from infusion to increase of neutrophil above >500/microL.

**Table 3:** Complications during each first and second HDC/ASCT.

Complication	1st HDC/ASCT	2nd HDC/ASCT			
	TTC (n = 33)	MEC (n = 20)	mMEC + ^131^I-MIBG (n = 13)	*p*-Value	Total (n = 33)
TRM (%)		1 (5.0)	1 (7.7)	1.00	2 (6.1)
VOD (%)	0 (0.0)	1 (5.0)	0 (0.0)	1.00	1 (3.0)
TMA (%)	0 (0.0)	1 (5.0)	1 (7.7)	1.00	2 (6.1)
Acute toxicity, CTCAE Grade 3/4					
Febrile neutropenia (%)	33 (100)	19 (95.0)	13 (100)	1.00	33 (97.0)
Pericardial effusion (%)	0 (0.0)	1 (5.0)	0 (0.0)	1.00	1 (3.0)
Diarrhea (%)	13 (39.4)	3 (15.0)	1 (7.7)	1.00	4 (12.1)
Vomiting (%)	10 (30.3)	3 (15.0)	1 (7.7)	1.00	4 (12.1)
Oral mucositis (%)	22 (66.7)	8 (40.0)	7 (53.8)	0.435	15 (45.5)
Total bilirubin (%)	0 (0.0)	1 (5.0)	1 (7.7)	1.00	2 (6.1)
LFT elevation (%)	15 (45.5)	19 (95.0)	1 (7.7)	<0.001	20 (60.6)
AKI (%)	0 (0.0)	1 (5.0)	1 (7.7)	1.00	2 (6.1)
Creatinine (%)	0 (0.0)	2 (10.0)	1 (7.7)	1.00	3 (9.1)
Proteinuria (%)	0 (0.0)	3 (15.0)	1 (7.7)	1.00	4 (12.1)
Hematuria (%)	2 (6.1)	0 (0.0)	1 (7.7)	0.394	1 (3.0)
Long-term toxicity (n = 26)		(n = 17)	(n = 9)		(n = 26)
Secondary malignancy (%)		4 (23.5)	0 (0.0)	0.263	4 (15.4)
Hypothyroidism (%)		2 (11.8)	2 (22.2)	0.591	4 (15.4)
Growth failure (%)		6 (35.3)	2 (22.2)	0.667	8 (30.8)

TRM: therapy-related mortality, VOD: Veno-occlusive disease, TMA: Thrombotic microangiopathy, t-MDS: therapy-related myelodysplastic syndrome, LFT: Liver function test, AKI: Acute kidney injury.

## Data Availability

The data presented in this study are available upon request from the corresponding author. The data are not publicly available because of the institutional privacy policies.
